# Keyword Spotting Using Human Electrocorticographic Recordings

**DOI:** 10.3389/fnins.2019.00060

**Published:** 2019-02-19

**Authors:** Griffin Milsap, Maxwell Collard, Christopher Coogan, Qinwan Rabbani, Yujing Wang, Nathan E. Crone

**Affiliations:** ^1^Department of Biomedical Engineering, Johns Hopkins University, Baltimore, MD, United States; ^2^Department of Neurology, Johns Hopkins University School of Medicine, Baltimore, MD, United States; ^3^Department of Electrical Engineering, Johns Hopkins University, Baltimore, MD, United States; ^4^Fischell Department of Bioengineering, University of Maryland College Park, College Park, MD, United States

**Keywords:** electrocorticography (ECoG), keyword spotting (KWS), automatic speech recognition (ASR), brain computer interface (BCI), speech, sensorimotor cortex (SMC), superior temporal gyrus (STG), articulation

## Abstract

Neural keyword spotting could form the basis of a speech brain-computer-interface for menu-navigation if it can be done with low latency and high specificity comparable to the “wake-word” functionality of modern voice-activated AI assistant technologies. This study investigated neural keyword spotting using motor representations of speech via invasively-recorded electrocorticographic signals as a proof-of-concept. Neural matched filters were created from monosyllabic consonant-vowel utterances: one keyword utterance, and 11 similar non-keyword utterances. These filters were used in an analog to the acoustic keyword spotting problem, applied for the first time to neural data. The filter templates were cross-correlated with the neural signal, capturing temporal dynamics of neural activation across cortical sites. Neural vocal activity detection (VAD) was used to identify utterance times and a discriminative classifier was used to determine if these utterances were the keyword or non-keyword speech. Model performance appeared to be highly related to electrode placement and spatial density. Vowel height (/a/ vs /i/) was poorly discriminated in recordings from sensorimotor cortex, but was highly discriminable using neural features from superior temporal gyrus during self-monitoring. The best performing neural keyword detection (5 keyword detections with two false-positives across 60 utterances) and neural VAD (100% sensitivity, ~1 false detection per 10 utterances) came from high-density (2 mm electrode diameter and 5 mm pitch) recordings from ventral sensorimotor cortex, suggesting the spatial fidelity and extent of high-density ECoG arrays may be sufficient for the purpose of speech brain-computer-interfaces.

## 1. Introduction

Keyword spotting (KWS) has recently come to the forefront of human-computer-interaction with the advent of voice-assist technologies such as Amazon Alexa, Apple's Siri, and Google's Assistant. All of these systems employ local, low-resource acoustic keyword search in real-time to detect a “wake word” that activates server-side speech recognition for interaction with an intelligent agent. These systems have been commercially successful and lauded for their ease of use. There are scenarios where voice-activated system interaction is suboptimal, especially when many speaking voices make the acoustic speech recognition less reliable and socially awkward to use. The ability to trigger an intelligent agent or perform menu selections with low latency and high specificity using neural control is of great practical interest.

A number of studies of neural speech decoding motivate the selection of electrocorticography (ECoG) for neural keyword spotting. Bouchard et al. (2013) were the first to examine the organization of articulation in ventral sensorimotor cortex (vSMC) using high-density ECoG recordings. Their study revealed that high frequency activity in the high-gamma range (70–110 Hz) encodes precise movements of speech articulators with a high degree of temporal specificity. Mugler et al. (2014, 2015) similarly characterized the articulatory representation in this area and further showed that this activity is more related to the gestural trajectories of specific muscles in the vocal tract than it is related to the specific keywords or phonemes articulated. Kanas et al. (2014) used high frequency content of speech-active areas of the brain to perform voice-activity-detection, or VAD—segmenting periods of speech from non-speech periods. Moreover, high-gamma activity from ECoG arrays was used as input to a language model and a small-vocabulary continuous speech recognition from neural signals was created in a study by Herff et al. (2015). Decoding of phonemic (Pei et al., 2011a; Bouchard and Chang, 2014) and gestural (Lotte et al., 2015; Mugler et al., 2015) content from vSMC has repeatedly been shown as well. These studies provide evidence that the dynamics of speech require the spatiotemporal resolution of intracortical electrophysiological recordings; features derived from non-invasive modalities do not modulate at rates necessary to make short-time inferences about articulatory processes. This study employs subdural ECoG recordings to determine the feasibility of neural keyword spotting using high quality neural recordings as a proof of concept.

In building a neural keyword spotter, we were inspired by acoustic keyword spotting, where this has been accomplished in a variety of ways. Hidden Markov Models (HMM) have been applied to this problem extensively. HMM based real-time keyword spotting tends to use a silent state, a keyword state (or series of states) and a set of “garbage” states that capture typical non-keyword speech. In “whole-word” approaches, each state of the HMM represents an entire word (Rohlicek et al., 1989; Rahim et al., 1997), whereas phonetic-based approaches (Rohlicek et al., 1993; Bourlard et al., 1994; Manos and Zue, 1997) break down the keyword and non-keyword utterances into sequences of phoneme sub-models. A keyword has been identified in the window of interest if the state sequence prediction proceeds through a keyword state (for whole-word modeling) or sequence of phonetic states corresponding to a keyword. Using a phonetic-based model to perform neural keyword spotting is risky: according to Mugler et al. (2014), a full set of American English phonemes has only been decoded at 36% accuracy from implanted ECoG arrays, motivating a whole-word approach.

Keshet et al. (2009) suggested a low-latency acoustic keyword spotting using a discriminative approach rather than a HMM-based probabilistic model. In this approach, a linear classifier is trained to maximize the margin between acoustic feature sequences containing keywords and others that don't. As detailed in the aforementioned study, this approach does not rely on computationally intensive Viterbi decoding and achieves higher keyword spotting performance than HMM-based systems.

**Figure 1:**
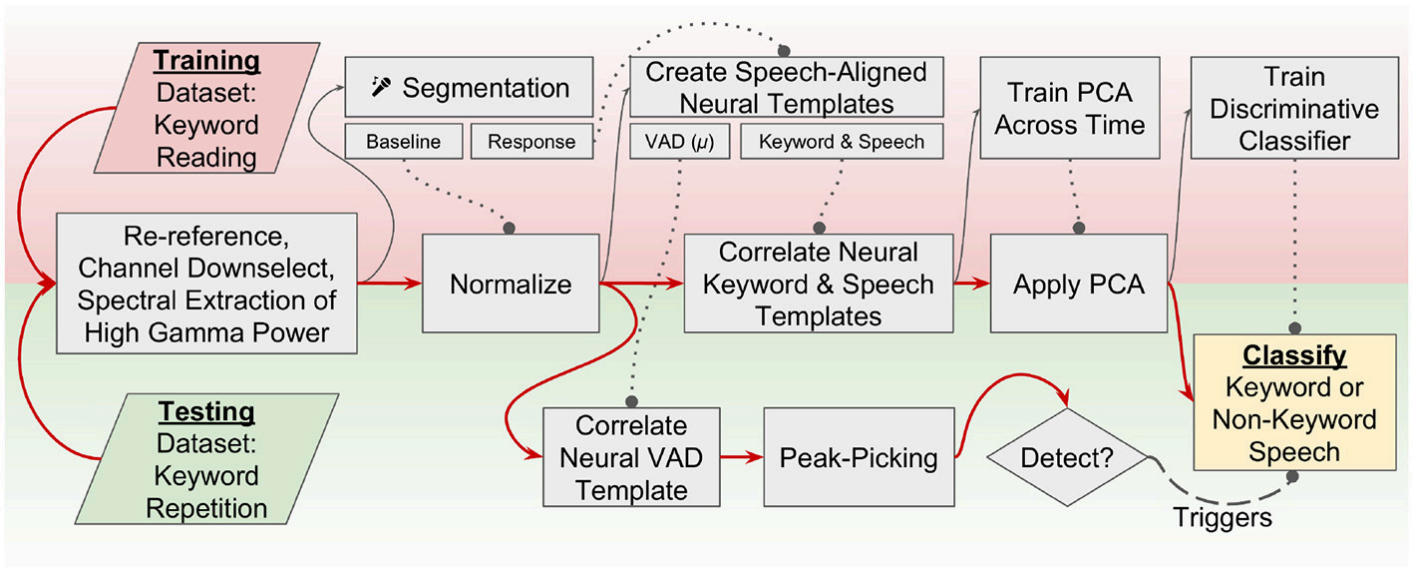
A flowchart representation of the keyword-spotting signal processing pipeline. Red arrows indicate flow of data through the pipeline. Dotted lines with circles indicate models that were trained on the training dataset are used in this step for both the training and testing data. Training is performed using a visually presented keyword reading paradigm, and testing occurs across an auditory keyword repetition task. This study implements a two-stage detector; one neural VAD template is correlated across the testing dataset, and peak-picking indicates a detected utterance. When an utterance is detected, a discriminative classifier is used to decide if the utterance was a keyword or non-keyword speech. Channel downselection, normalization parameters, neural templates, feature dimensionality reduction, and classifiers are all trained on the reading (training) task and applied to the repetition (testing) task to simulate how keyword spotting would realistically perform in a separate recording session.

We have chosen to use a neural voice-activity detection combined with an adaptation of the aforementioned discriminative (non-HMM-based) approach to perform neural keyword spotting. A flowchart that describes the signal processing chain and two-step discriminative decoding pipeline is described in Figure 1. Application of neural features to existing acoustic KWS approaches requires a few modifications. For example, mel-frequency cepstral coefficients derived from a single spectrally-rich microphone recording are sufficient to perform acoustic keyword recognition; by contrast, there are many electrodes in an ECoG recording, each with a single time-varying “activation” signal, corresponding to changes in neural population firing rates, in turn indexed by changes in high frequency activity. These activations capture neural processes necessary to sequence, control, and monitor the production of speech, as opposed to acoustic features that capture discriminable aspects of spoken acoustic waveforms. The motor representations of speech that capture the dynamics of articulators, and the auditory representations of speech that capture phonetic content during self-monitoring but also activate during perceived speech, are of particular interest to a neural keyword spotting system.

A recent study by Ramsey et al. (2017) has significantly influenced the approach we've developed to capture the spatiotemporal dynamics of neural features for the purpose of informing keyword discrimination. In the study, Ramsey discriminated phonemes from high density ECoG recordings of vSMC using the correlation of spatiotemporal matched filters as a means of identifying when the spatiotemporal pattern of high frequency activity matched stereotyped patterns for articulations (or gestural sequence of articulations). This method achieved 75% accuracy in a four-class phoneme discrimination problem, and highlighted the importance of including temporal relationships of high frequency activity between cortical sites in decoding models. We extend this methodology here to the creation of maximally discriminative “neural templates” to identify consonant-vowel “keyword” utterances instead of single phonemes.

“Wake-words” for voice-assist technologies are typically chosen to be low-frequency and phonetically complex to reduce the number of spontaneous detections. To simplify the problem of producing a more neurally detectable keyword, we examine monosyllabic, “consonant-vowel” keywords, varying the place of articulation, the consonant voicing, and the vowel height during phonation. Within the context of this study, any speech following the presentation (either textual or auditory) of a CV syllable stimulus is called an “utterance.” Keyword spotter models were trained/tested on one of those utterances, defined as the “keyword” utterance for that particular model, with the rest defined as non-keyword utterances—resulting in the creation of 12 keyword spotter models per subject; each sensitive to a specific monosyllabic keyword. We have chosen to examine keyword detection accuracy with respect to non-keyword speech and silence, as opposed to a multi-keyword decode to further simplify the problem and performance metrics. We will also limit ourselves to causal methods of feature extraction and classification for this study to realize how neural keyword detection would perform if deployed in a low-resource real-time scenario.

This is the first study of neural keyword spotting in ECoG recordings that demonstrates low latency (~1 s) using causal models and feature extraction methods akin to low-resource acoustic KWS implementations. Application of spatiotemporal matched filters, trained/tested in separate ECoG recordings, appeared to strongly influence the specificity of the spotter in single-trials. We found that spatial **and** temporal features from vSMC can be used to discriminate place of articulation and consonant voicing in monosyllabic keywords, and that vowel height (/a/ vs /i/) is much more discriminable using neural features from STG during self-monitoring of overtly produced speech, as opposed to the motor representations that are simultaneously present in vSMC.

## 2. Materials and Methods

Please refer to Figure 1 for a brief overview of the methodology used in this study, summarized here for convenience, but with more detail provided in subsections 2.1-2.6. Two datasets were collected in separate recording sessions; one used for training model parameters and another used for performance validation. The first step in the signal processing pipeline was a signal re-referencing to the common average followed by a manual channel downselection and spectral extraction of high gamma log-power. High gamma modulation across the training task was normalized (z-scored) per-channel to a pooled baseline period constructed by segmenting baseline periods from across the training task. Response periods in the training dataset were used to train neural templates, and these templates were cross-correlated over the normalized high gamma features before a principal component analysis (PCA) was fit. Discriminative classifiers for each keyword were fit on these “template-PC” features and a decision boundary was chosen for each keyword spotter individually. Similarly, a threshold parameter was selected for a causal peak-picking algorithm applied to the cross-correlation of the grand-average “VAD” template. The same preprocessing steps were applied to the testing dataset, and high gamma log-power was normalized across testing dataset. The templates and PCA that were trained previously were then applied without further calibration to the testing high gamma features, and template-PC features corresponding to super-threshold peaks in the VAD template output were classified using the aforementioned discriminative classifiers.

### 2.1. Data Collection

**Figure 2:**
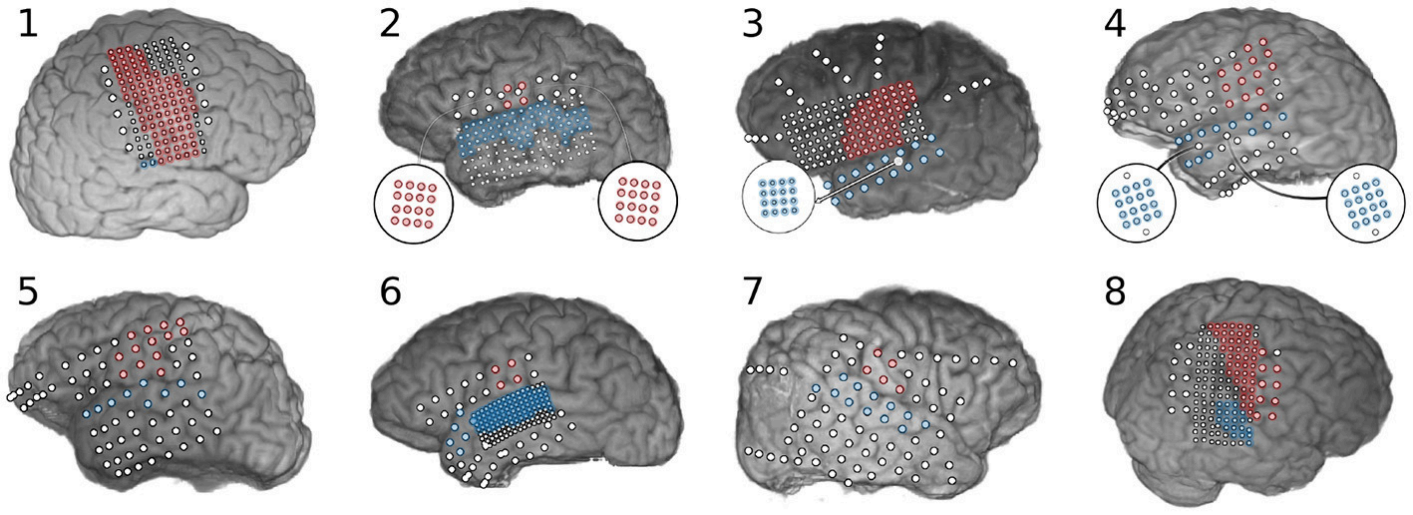
Neuroimaging and electrode localization for eight subjects implanted with subdural electrode arrays. Electrodes positioned over sensorimotor cortex are highlighted in red and electrodes over superior temporal gyrus are highlighted blue. Biographical and experimental details for these subjects can be found in Table 1. Subject 1 had a large lesion within pre-central gyrus, from which very little high frequency activity was recorded. Subject 3 had an ictal locus very near sensorimotor cortex with substantial inter-ictal activity that limited observation of neural features in this area. Subject 8 had a lesion in the right supramarginal gyrus.

Subdural electrocorticographic recordings were made in eight subjects undergoing intracranial monitoring prior to resective surgery for drug-resistant epilepsy. Electrocorticographic (ECoG) arrays of platinum electrodes with varying exposed area and spatial density were placed for a 1–2 week period according to clinical requirements. Subjects performed both syllable-reading and syllable-repetition paradigms as part of a protocol approved by the Johns Hopkins University Institutional Review Board. All subjects gave written informed consent in accordance with the Declaration of Helsinki. Electrode localization was performed by aligning electrode locations from a post-operative computed tomography image with a pre-operative magnetic resonance image using Bioimage Suite (Papademetris et al., 2006). Neuroimaging and electrode locations are shown in Figure 2.

Subjects performed two tasks wherein they were asked to overtly produce monosyllabic consonant-vowel (CV) utterances. In the (syllable) reading task, a textual representation of the utterance was visually presented for 1 s (see **Table 2** for details) followed by an intertrial interval of 2–3 s during which the subject was instructed to fixate on a visible fixation cross. The (syllable) repetition paradigm was identical, except that the fixation cross remained on screen throughout the task and the utterance was aurally cued using a speaker. In both tasks, the subject was instructed to speak the prompted syllable aloud after stimulus delivery, and a microphone was used to record the subject's responses to a high quality digital audio file. A monitor-output cable connected the microphone recording device (Zoom H2, Zoom Corporation, Tokyo, Japan) to an auxiliary analog input on the electrophysiological amplifier (Neuroport, Blackrock Microsystems, Salt Lake City, UT; and EEG1200, Nihon Kohden, Tomioka, Japan), recording a lower-resolution version of the subject's speech synchronized with the ECoG data at 1,000 samples per second. BCI2000 (Mellinger and Schalk, 2007) was used to present stimuli and record the data from the amplifier into a standardized format for offline analysis. Data was collected in blocks of 60 trials; 5 trials each for all 12 utterances in a randomized order. The paradigm was split across two blocks of reading and two blocks of repetition for each subject, but time and clinical constraints limited collection to one-block of the tasks for some subjects. Details of data collection for each subject is documented in Table 1.

**Table 1:** Biographical and experimental details for subjects.

**ID**	**Side**	**Age**	**Sex**	**Reading trials**	**Repetition trials**	**Grid specifications**

1	R	17	M	120	60^*^	vSMC: 85 (85 HD-5) STG: 2 (2 HD-5) Total: 87
2	L	37	F	60	120	vSMC: 36 (32 μ, 4 SD) STG: 57 (57 HD-5) Total: 93
3	L	25	M	105^**^	120	vSMC: 30 (30 HD-5) STG: 32 (16 μ, 16 SD) Total: 62
4	L	39	M	120	120	vSMC: 14 (14 SD) STG: 48 (32 μ, 16 SD) Total: 62
5	L	40	M	120	120	vSMC: 13 (13 SD) STG: 9 (9 SD) Total: 22
6	L	40	F	60	120	vSMC: 4 (4 SD) STG: 87 (81 HD-3, 6 SD) Total: 91
7	R	27	M	120	60	vSMC: 5 (5 SD) STG: 12 (12 SD) Total: 17
8	R	19	M	120	120	vSMC: 52 (43 HD-5, 9 SD) STG: 19 (HD-5) Total: 7

The implant hemisphere (side), age, sex, number of reading/repetition trials, and grid specifications for all eight subjects in the study are listed here. Associated neuroimaging and electrode localization can be found in Figure 2. Channels are delineated by region of interest, and further by the diameter of the electrode's exposed area, then by the inter-electrode spacing. SD: Standard macro-array (2 mm diameter, 1 cm pitch). HD-5: High density array (2 mm diameter, 5 mm pitch). HD-3: High density array (1 mm diameter, 3 mm pitch). μ: Micro-ECoG array (75 μm diameter, 1 mm pitch).

* 120 trials were recorded, but the synchronized microphone recording failed for the second set of 60 trials. Neural keyword spotting can be applied to this second block, but ground truth timing metrics are unavailable.

** *Recording session ended early*.

### 2.2. Preprocessing and Segmentation

Seventy one channels across all subjects (1–18 per subject) were identified as noisy/bad by a neurologist via visual inspection of the raw ECoG signals and were removed from further analysis. Spatial filters were applied to re-reference recordings to the common-average of the included channels. Trial markers from BCI2000 that designated stimulus presentation (auditory or visual) were used to define the trial onset points. The 250–450 Hz band-power in the synchronized low-fidelity microphone recording captured the first formant of speech in each subject, and was thresholded to detect the voice onset time for each trial. These threshold crossings tend to be associated with the voice-onset-time in CV keywords containing a voiced consonant and the plosive release in CV keywords containing an unvoiced consonant, due to the silent nature of consonant articulation. Templates were generated from a 1-s “response” period centered around this threshold crossing to capture differences in the timing of neural features relative to the response onset (Mugler et al., 2014; Jiang et al., 2016; Ramsey et al., 2017). Neural features were normalized within each task individually to a pooled “baseline” period which was created from a 1-s period prior to stimulus presentation across all trials within a single task. All trials from the reading dataset were used for training templates and classifiers that were applied across the repetition dataset. In this way, the training data were entirely separate from the testing data, and the templates generalized feature extraction across tasks.

### 2.3. Feature Extraction and Electrode Downselection

Electrodes over sensorimotor cortex and superior temporal gyrus were manually identified by a neurologist; see Figure 2 for a summary. Electrodes lying outside these areas were excluded from further analysis. A 128 ms window sliding by 16 ms increments was used to perform spectral decomposition via the fast Fourier transform. Spectral power was log-transformed and z-scored to the baseline period, per-frequency. Frequency bins between 70 and 110 Hz were averaged together to form a time-varying feature capturing the band power modulations in the “high-gamma” range, a frequency range highly correlated with the firing of local neural populations (Ray et al., 2008). This feature was then re-normalized to the baseline period per-electrode.

### 2.4. Template Generation and Voice Activity Detection

Previous studies indicate that the timing of high gamma activity contributes significantly to decoding of speech from vSMC (Jiang et al., 2016; Ramsey et al., 2017). Neural templates were trained to capture spatiotemporal relationships of high gamma activity in an efficient, but causal representation. A “response template” was created by calculating the mean of the neural responses from all trials (*N* = 60–120) in the training dataset. A “keyword template” for each keyword was also created by calculating the mean of the neural responses for each of the keywords individually (5–10 trials). We additionally took advantage of our keyword design to create neural templates composed of higher trial counts across axes of articulation, as described in Table 2. The response template was then subtracted from each of these keyword templates, the resulting “discrimination template” captured spatiotemporal relationships that differed from the mean neural responses in the response template. A significance mask was created by z-scoring the condition mean (prior to subtraction of the response template) relative to the baseline period. A temporal smoothing kernel (hamming, 0.1 s) was applied to reduce noise in the template before the significance mask was applied; elements with a z-score of <3.0 were set to zero to further reduce noise. The smoothed and regularized discrimination templates were correlated with the corresponding high-gamma features in both testing and training datasets—these features were further smoothed (hamming, 0.25 s) to reduce the influence that slight timing mismatches could have on keyword discrimination. An example visualization of the generation of a discrimination template for bilabial keywords can be found in **Figure 4A**. A PCA was fit to identify linear combinations of template output features that accounted for 90% of the variance across the entire reading task. Principle components of template outputs were calculated for both the reading and repetition datasets, reducing covariance in the template outputs and creating neural features which can be used for keyword discrimination.

**Table 2:** Utterances and associated axes of articulation.

**/IPA/ (“Stim”)**	**Bilabial**	**Alveolar**	**Velar**
Voiced	/ba/ (“BAH”)	/da/ (“DAH”)	/ga/ (“GAH”)
Unvoiced	/pa/ (“PAH”)	/ta/ (“TAH”)	/ka/ (“KAH”)
**/IPA/ (“Stim”)**	**Bilabial**	**Alveolar**	**Velar**
Voiced	/bi/ (“BEE”)	/di/ (“DEE”)	/gi/ (“GEE”)
Unvoiced	/pi/ (“PEE”)	/ti/ (“TEE”)	/ki/ (“KEE”)

*Utterances vary on three axes; three places of articulation, two ways of consonant voicing, and two vowel heights. Utterances are shown with their IPA notation as well as the textual prompt as shown in the reading paradigm. “GEE” would typically be pronounced /Ãi/ but subjects were instructed to respond with /gi/ instead*.

Electrodes from STG were excluded from the response template; the resulting template was used as the neural VAD template. Auditory representations of speech in STG tend to have less specificity to self-generated speech and their inclusion in the VAD model can result in false-positive detections coincident with the *perception* of speech, whether or not it was produced by the subject. Neural VAD was calculated as the squared temporal correlation between the VAD template and the normalized high-gamma power. VAD output was further smoothed using a temporal smoothing kernel (hamming, 1.0 s). A causal peak-picking algorithm was applied to identify utterance onset times—the derivative of the neural VAD signal was thresholded and the zero-crossing that follows a threshold crossing was chosen as the utterance detection time. Example templates and their corresponding correlational output are shown in **Figure 4**. Application of these templates to live neural features results in exactly 1 s of latency for neural VAD and keyword discrimination.

### 2.5. Discriminative Classification

A discriminative classifier similar to SVM, as described in great mathematical detail by Keshet et al. (2009), was trained on the reading dataset. In broad strokes, the training step attempted to designate a linear discrimination boundary that maintains a constant margin of separation between pairs of feature-vectors corresponding to keyword and non-keyword utterances. For each pair, the training step searched for the feature-vector within ±100 ms of the alignment time for the non-keyword utterance that looked maximally “keyword-like,” given the current discrimination boundary. The learning step adjusted the discrimination boundary using the difference between that maximized non-keyword feature-vector and the ground-truth keyword feature-vector. A significant advantage of this classifier is that it can be trained online as new observations become available.

Pairs of feature-vectors associated with keyword and non-keyword utterances were assigned within stimulus blocks. Additionally, feature-vectors associated with keyword utterances were paired with feature-vectors corresponding to silent periods (1.0 s before stimulus onset) to adapt the classifier boundary to VAD false-detections during silent periods. In **Figure 5**, classifier output was calculated using ground-truth utterance detections derived from the microphone. During simulated testing, results of which are shown in **Figure 6**, the classifier output was calculated at times when the neural VAD model identified an utterance. The slight temporal misalignments between neural VAD and microphone-derived timing accounts for the different classifier performances between these figures.

### 2.6. Testing and Performance Metrics

The templates, principle components, and discriminative classifiers were trained on all trials of the reading task. Testing and performance metrics were calculated from the application of these models to the repetition task. A VAD performance metric was calculated by sweeping the aforementioned VAD threshold value from 0 to 20 standard deviations (relative to baseline periods) and comparing the utterance detection times to the ground-truth microphone threshold crossings. An utterance detection within ±100 ms of a microphone event was classified as a true-positive, but subsequent detections for that utterance were considered false-positives.

An ROC curve was created for each of the keyword classifiers using microphone-derived voice onsets in the repetition task. A classifier threshold was swept from −10 to 10 and the resulting keyword detections and false-positives were used to create an ROC curve and derive area-under-curve (AUC) metrics for each keyword classifier. Significance of the AUC statistic was calculated by scrambling the ground-truth utterance labels while training keyword detectors. A bootstrapped null-distribution of 1000 AUC metrics was generated for each keyword classifier, from which statistical significance thresholds for the metric were calculated. Keyword spotting performance using neural VAD times was also calculated for each classifier using a threshold that was chosen to maximize sensitivity while minimizing false detections' in particular, equalizing the error rates for false-negatives and false-positives, the so-called “equal error rate” condition (Motlicek et al., 2012)—on the training dataset.

## 3. Results

**Figure 3:**
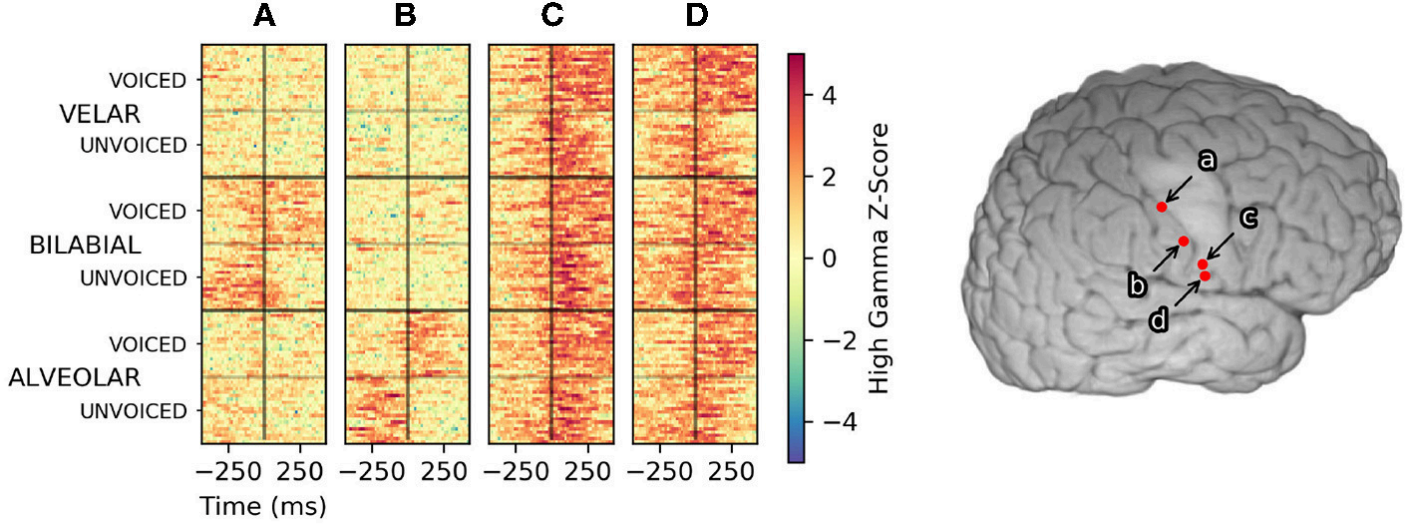
High-gamma single-trial rasters across the reading task from four manually selected electrodes in Subject 1. Trials, plotted along the Y axis, were sorted first by the place of articulation for the consonant, then by consonant voicing. Trials were aligned with response-onset time set to 0 s, denoted by a black vertical line at the center of each raster. Color denotes the high-gamma feature z-score normalized to a pooled pre-trial baseline period. Activity in electrode **(A)** appears to represent a bilabial place of articulation, whereas activity in electrode **(B)** appears to indicate an alveolar place of articulation. Timing differences of high-gamma activity relative to the voice onset time encoded the voicing of bilabial and alveolar consonants in these areas. Electrode **(C)** exhibited consistent high-gamma amplitude and timing for all utterances; informing neural VAD but less useful for keyword discrimination. Electrode **(D)** appeared to encode consonant voicing across all places of articulation. No clear patterns emerged if the trials were sorted by vowel height (/a/ vs /i/) for any electrodes in Subject 1.

Within the context of this methodology, discrimination between keyword and non-keyword speech relies upon differences in timing and/or amplitude of high-gamma activity. Differences in high-gamma amplitude across keywords are useful in traditional decoding approaches where only single time-points of high-gamma activity are used to make classification decisions. Single-trial plots, as seen in Figure 3, suggest high-gamma amplitude in vSMC can be sufficient to decide the place-of-articulation for an utterance. Consonant voicing appears to be encoded in the timing of high-gamma activity relative to voice onset time. The sensation of pressure build-up in the vocal tract prior to plosive release is a plausible explanation for the timing of this discriminable neural activation in electrodes **a** and **b**, especially given the placement of these electrodes in postcentral gyrus; an area typically associated with sensation.

**Figure 4:**
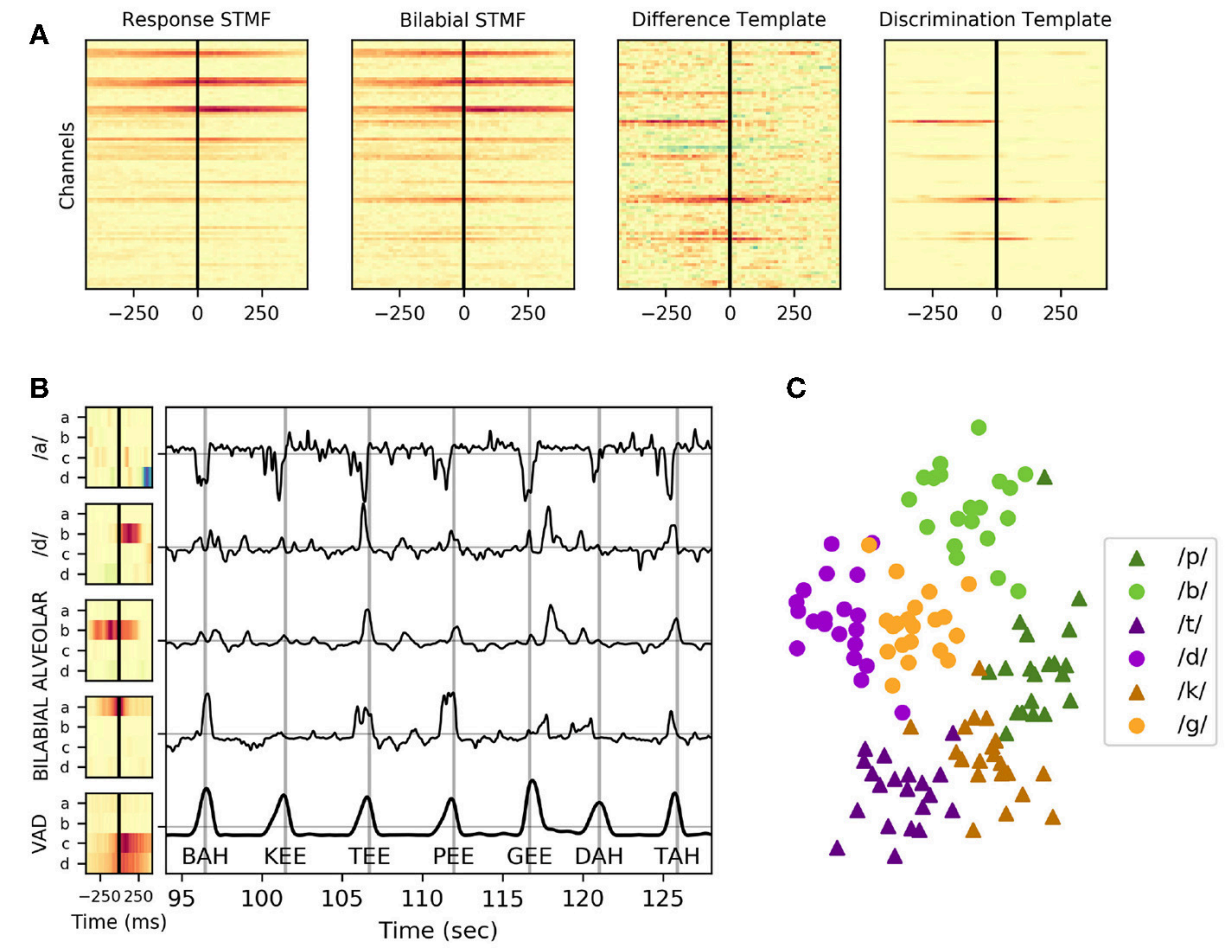
Example neural templates and utterance discrimination in Subject 1. **(A)** From left to right: the response spatiotemporal matched filter; an average of all keyword utterances, the bilabial spatiotemporal matched filter (STMF); an average of just keyword utterances with a bilabial place of articulation, the difference template; the subtraction of the response spatiotemporal matched filter from the bilabial spatiotemporal matched filter, and the discrimination template; the regularized and smoothed/denoised discrimination template for bilabial keywords. **(B)** Neural templates, created as a trial-average of particular keywords or phonemic contrasts followed by regularization and normalization, are shown for the four electrodes (**a**, **b**, **c**, and **d**) depicted in Figure 3. The VAD template, shown at the bottom, is the mean across all 120 trials in the task. The correlation of these templates with the high-gamma activity in the same task is shown in the plot to the right of the templates for a contiguous period of ~95 to ~125 s into the reading task. Vertical gray lines in this plot indicate ground truth utterance times as recorded by a microphone, and the associated utterance is indicated at the bottom of these lines. Peaks of the neural VAD output closely matched the utterance times. **(C)** The values of these template features across all templates (including many not pictured) at the utterance onset times were collected and reduced to two dimensions using multi-dimensional scaling, then plotted in the scatter plot, highlighting how these features clearly discriminate place of articulation and consonant voicing.

The correlation of neural templates with high-gamma activity created high-level features that appeared to be useful for clustering utterances using these spatiotemporal relationships, as shown in Figure 4. The discriminative quality of a neural template appeared to rely primarily upon the number of trials used to create it; a decrease in the template noise was associated with a higher number of trials. A neural template for a particular contrast highlights the difference from the mean template, which can be a problem if there is no discriminable difference between the contrasts. As seen in Figure 4, Subject 1 had very little discriminable activity within the vowel height condition (/a/ vs /i/), meaning the trial average across the ‘/a/’ condition and the ‘/i/’ condition were very similar to the trial grand-average. Subtracting the trial-average from the two condition averages resulted in a template that introduced significant noise to the feature set. The inclusion of these templates was less of a problem due to the following decomposition of these features into principal components; the noisy template outputs tended to be de-emphasized as they did not explain much of the variance of the features across time. Noting that template output appeared to fluctuate around neural VAD timings, temporal alignment was absolutely critical when interpreting these features.

**Figure 5:**
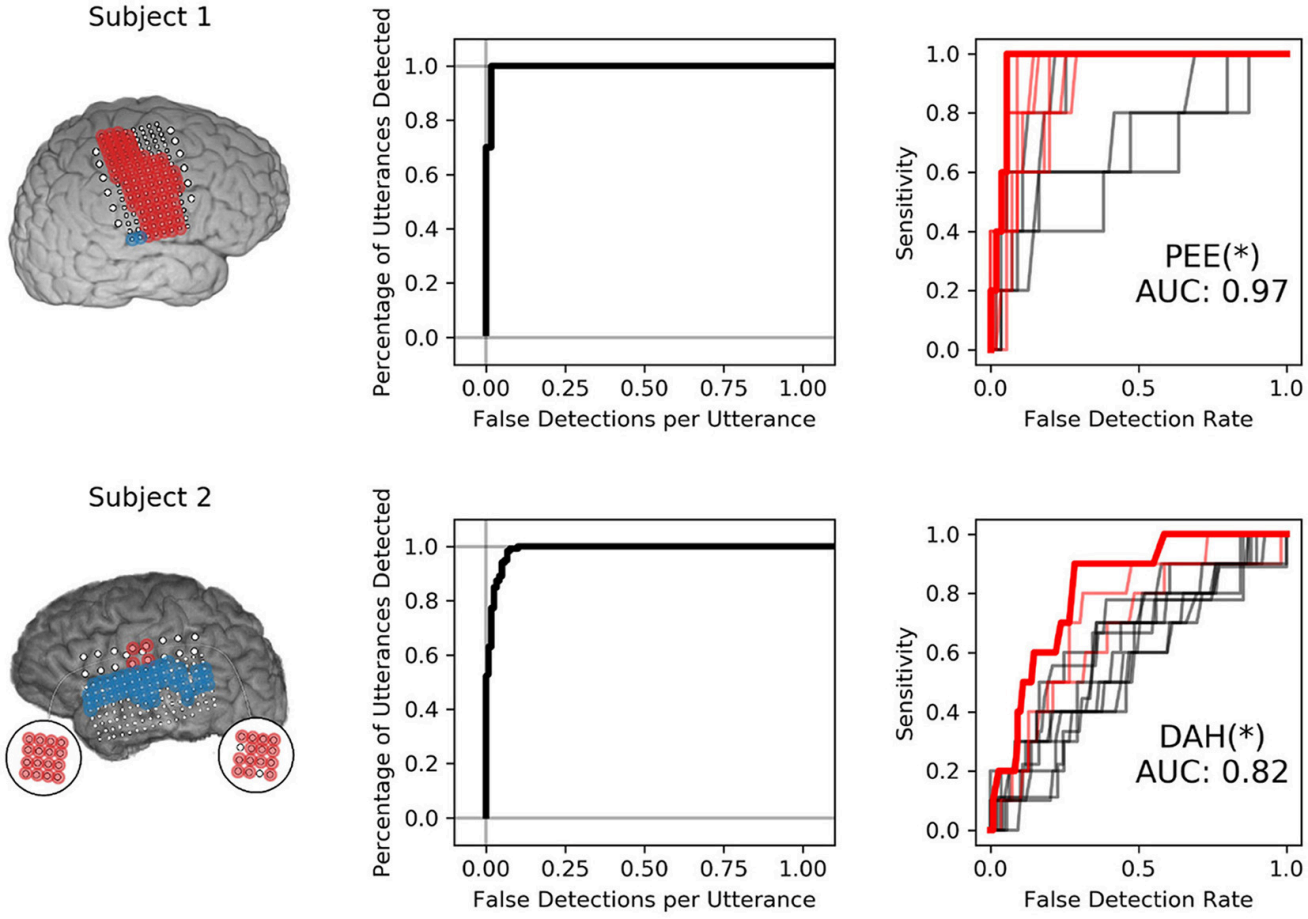
Isolated VAD and keyword discriminability for two subjects. The left-most panel shows the electrodes highlighted in red and blue that were used to discriminate syllables. Only electrodes highlighted in red were used to perform VAD. The center panel shows the VAD performance in sensitivity (percentage of utterance timings correctly identified) against the number of false detections per utterance for various VAD thresholds. The right-most panel shows ROC curves for all 12 keyword detectors. ROC curves with AUC values were significant at the 95% confidence interval are highlighted in red. The keyword detector that produced the highest AUC is highlighted in bold-red and indicated via annotation under the curves, followed by an asterisk that indicates significance at the *p* < 0.05 level with respect to the distribution of maximum AUC models.

Neural VAD and keyword discriminability appeared to be somewhat decoupled; several subjects showed consistent high-gamma modulation across utterances that was useful for performing VAD, but these features were less useful for keyword discrimination, as shown in Figure 5. Subject 1 exhibited exceptional VAD with highly significant discrimination of several keywords. Subject 2 showed similar VAD performance, but demonstrated relatively poor keyword discrimination. Classifiers in Subject 1 leveraged neural features that discriminated consonants well (shown in Figure 3), whereas classifiers from Subject 2 were only informed by features that discriminated vowel height and alveolar place of articulation, shown in **Figure 7**. VAD and keyword discrimination results for all subjects are shown in Supplementary Material.

## 4. Discussion

This study is the first to examine keyword spotting using ECoG. A neural keyword spotter could form the basis of a menu-selection BCI for disabled users, or a low latency “neural click” in a virtual reality context where the user is unable to see/use a real-world input device. A BCI-enabled keyword spotter could respond selectively to the user whereas acoustic keyword spotters struggle to operate in multi-speaker conditions. These results were obtained by performing a two-step classification procedure involving neural VAD and keyword vs. non-keyword-speech classification. As mentioned previously, neural voice activity detection has been performed before using spectral decomposition techniques and a discriminative classifier by Kanas et al. (2014). Performing VAD using this method of template-based “matched filtering” has a number of benefits over this prior work. Due to the fact that all utterances are roughly the same length and surrounded by silence, cross correlation with the neural VAD template actually provides a good alignment point for the application of a discriminative classifier. Furthermore, the cross correlation is computationally efficient and only relies on a peak-picking implementation to find utterances. The second-stage discriminative classifier tends to classify VAD false detections as “non-keyword utterances,” and serves as a secondary filter before detecting keyword events.

**Figure 6:**
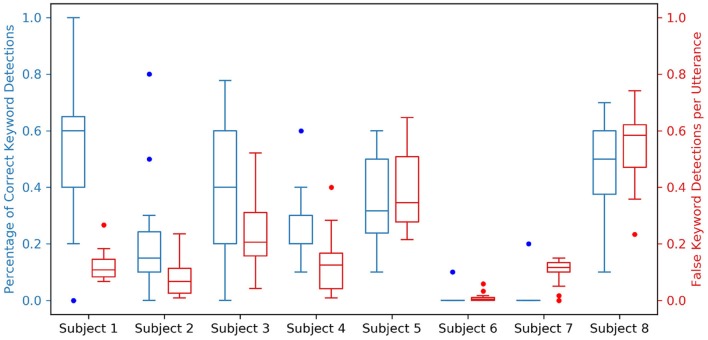
Simulated keyword spotting performance on the testing dataset for all spotters across all subjects. For each subject, two boxplots relating the performance of all 12 keyword spotters are shown; a blue boxplot to the left indicating the percentage of correct keyword detections with a value ideally closer to 1.0, and a red boxplot to the right indicating the percentage of false keyword detections per utterance with a value ideally closer to 0.0. These performance metrics are coupled to neural VAD performance, which is shown in the Supplementary Material for all subjects.

Acoustic “wake word” spotting typically relies on keywords that are low frequency and dissimilar from typical non-keyword utterances, the most popular wake words being words/phrases like “Alexa,” “Hey Siri,” and “Okay Google.” In this study, monosyllabic keywords were chosen to examine what makes keywords more distinguishable neurally as opposed to acoustically. The utterances used in our experiment were exceptionally similar to each-other, varying only by 1–3 distinctive articulatory features. Indeed, a particularly important feature—keyword length—was the same across all utterances, making the keyword detection problem significantly more difficult. The simulated keyword spotting performance for all keyword spotters in Subject 1 is shown in Supplementary Figure, and the simulated keyword spotting summary performance metrics are shown for all subjects in Figure 6. While this performance is not comparable with the current state of the art in acoustic keyword spotting, neural VAD alone appears to provide a temporally precise 1-bit (silent vs. speech) BCI and the addition of keyword discrimination would allow the user to trigger the BCI while not restricting speech between intended triggerings.

**Figure 7:**
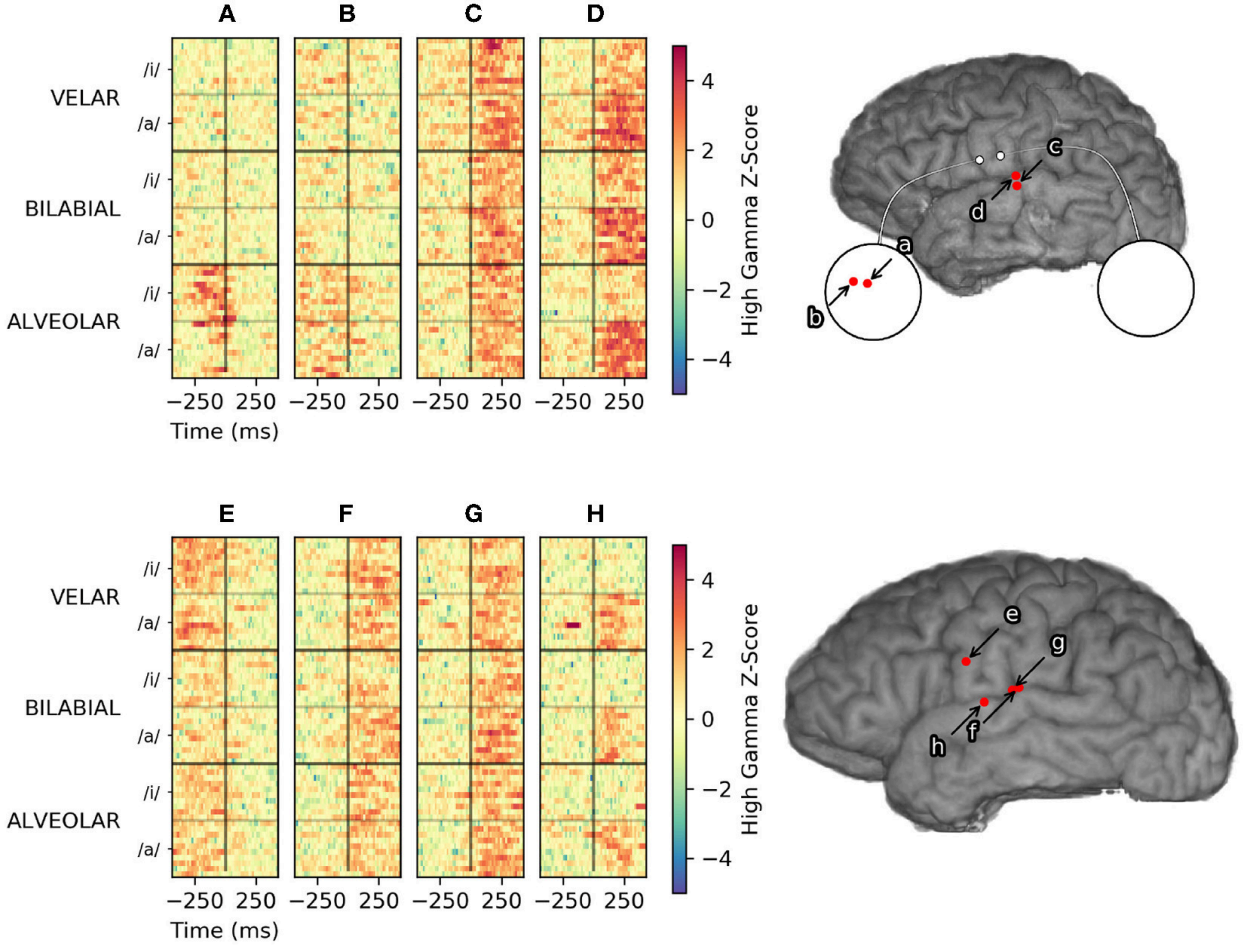
Vowel-specific high gamma activity from Subject 2 (top) and Subject 6 (bottom). Single-trial high-gamma rasters to the left are sorted first by place of articulation, then by vowel height. Electrode **(D)** and **(H)** appear to encode vowel height, in similar areas of STG. Electrode **(A)** and **(B)** are micro-ECoG electrodes over vSMC that appear to encode place of articulation. Electrodes **(C)**, **(F)**, and **(G)** appear to modulate consistently with all utterances and are more useful for VAD, but provide little discriminative information. Modulation recorded by electrode **(E)** appears to be consistently related to articulation, but does not appear to discriminate utterances by place of articulation or vowel height.

The most striking finding from this study was that vowel height was poorly represented in vSMC. This is consistent with the findings of Bouchard et al. (2013) in which syllable discrimination using a high-density grid in vSMC achieved lower cluster separability of vowel height than manner of consonant articulation. This result also corroborates a finding from Ramsey et al. (2017) that vowels are the least distinguishable phonemes in their test set; the authors speculated that lacking plosives, vowels differ only in lip positions, which may not be well-represented in this area. Our findings suggest that vowel height is well represented in auditory association cortex areas STG, presumably due to self-monitoring, shown in Figure 7.

None of the subjects in the study exhibited high gamma activity that significantly encoded vowel height within vSMC. Many studies indicate vowel phones may be decoded from vSMC (Pei et al., 2011a; Bouchard and Chang, 2014; Mugler et al., 2014; Ramsey et al., 2017), although some studies also note that decoding accuracy is generally worse than consonant phones (Mugler et al., 2014; Ramsey et al., 2017). None of the aforementioned studies report a failure to decode vowel phones from vSMC, which is contrary to our findings. This may be due to the fact that the vowels chosen for this study, /a/ and /i/, result from a slight variation in tongue height and do not involve differential activation of the lips, such as with the vowel contrasts selected for the aforementioned studies, /a/ and /u/, which can recruit sensorimotor areas related to the face. This said, STG has been shown to consistently modulate with differences in vowel height (Mesgarani et al., 2014) during audition and self monitoring. Practically, our results suggest that discrimination of vowels during keyword spotting with a neural interface may be improved by including auditory representations from STG with sensorimotor representations from vSMC. This finding also suggests that modulation and control of vowel height relies on interactions between auditory areas and motor areas more than consonant articulation which seems to be well represented in just suprasylvian cortex.

The subject with electrode coverage most analogous to the implant detailed in Bouchard et al. (2013) had a high-density grid with 2-mm electrode diameter and 5-mm interelectrode distance over somatosensory cortex. Although we showed no significant neural differences between low and high vowel height with this grid placement, the grid in Bouchard et. al. had a slightly smaller pitch and this higher resolution may have captured more information about vowel height than we observed. Similarly, we showed significantly worse performance with lower density coverage of vSMC, demonstrated by subjects with only standard-density (2 mm electrode diameter and 1 cm pitch) coverage, indicating that standard ECoG arrays are likely insufficient for a comprehensive speech neuroprosthesis. Some subjects were also implanted with microelectrode array grommets (75 μm electrode diameter and 1 mm pitch); these arrays have a sensor density similar to what is thought to be the spatial limit of subdural neural recordings (Slutzky et al., 2010). Micro-ECoG was useful in discriminating place of articulation for utterances from Subject 2, but its utility was greatly dependent on placement due to its limited spatial extent. An ideal ECoG array would probably cover all of vSMC with the same 1 mm pitch, but this is not yet technically feasible with clinically approved ECoG electrodes and their connectors. Although our best results came from a subject with a high density grid over vSMC, our inability to observe neural activity associated with velar consonants indicates that even these high density arrays do not capture sufficient detail to distinguish all articulators (and hence, all phones) necessary for a speech neuroprosthesis. Further research into recording devices that cover a similar spatial extent but with higher sensor density and channel counts might be fruitful, but our results indicate that neural features recorded from high density ECoG arrays can, at a minimum, produce a usable neural interface for whole-word keyword spotting in overt speech.

**Figure 8:**
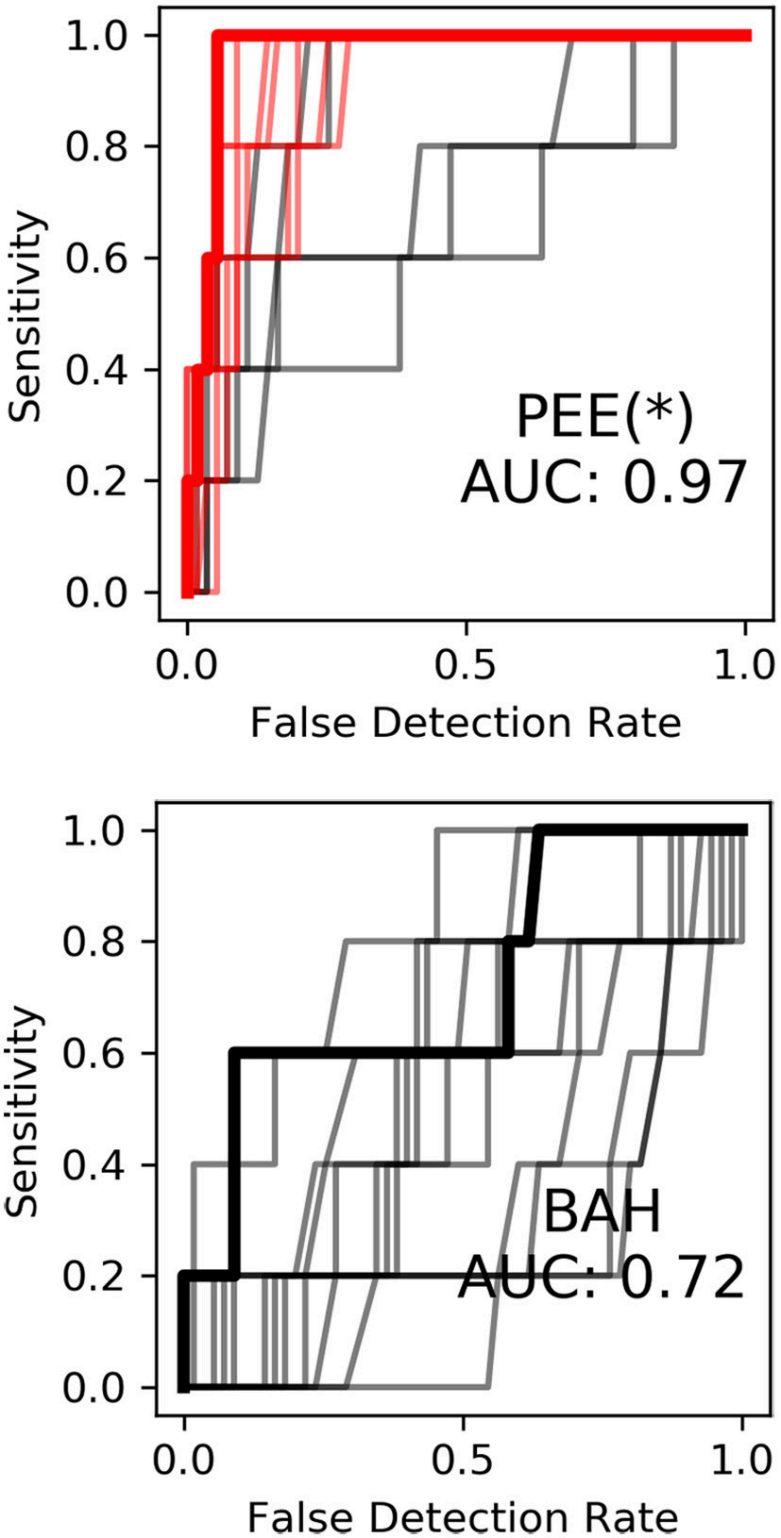
Keyword discrimination ROC curves for Subject 1 before (to the left) and after (to the right) replacement of neural templates with rectangular smoothing windows. Keyword discrimination performance dropped across all models suggesting inclusion of spatiotemporal relationships using neural templates aids keyword discrimination.

Correlating spatiotemporal templates with streaming high gamma features was primarily motivated by existing keyword search methodology, as well as a recent study by Ramsey et al. (2017). The temporal encoding of consonant voicing in Subject 1 (see Figure 3) further motivated the application of spatiotemporal template methodology. To evaluate the contribution of neural templates to keyword discrimination, the templates were replaced with a rectangular window of the same size, resulting in smoothing of the high gamma features on the same order as that of the templates. After making this change, we observed a marked drop in keyword discrimination, highlighted in Figure 8, suggesting that temporal relationships between high-gamma events provide information useful for discriminating keywords, and that these templates are an effective way of quantifying these relationships in single trials.

A rational application of neural keyword spotting would detect keywords that would have more contextual relevance in the presence of continuous speech. Commercial keyword spotting systems tend to select low-frequency words with more discriminable acoustic features that maximize the probability of keyword detection while minimizing the number of false-positive detections during typical non-keyword speech. The keywords chosen for this particular study were not selected with these considerations in mind, but rather highlight how keyword spotting performance varies primarily with the articulatory representations sampled. For Subject 1, there were no electrodes that showed high gamma modulation during the articulation of syllables with a velar place of articulation—yet within the context of this study—these articulations were readily discriminable *because* of this lack of neural activity. Within the context of Figure 3, a keyword that only modulated electrodes **c** and **d**, and not **a** or **b**, can be reasonably deduced to have a velar consonant, but if the keywords were downselected to just “GAH, KAH, GEE, and KEE,” these keywords would not be sufficiently discriminable using the coverage from Subject 1. As described earlier, Subject 1 also had no discriminable neural modulation across the vowel contrast in the keyword set, with false detections tending to trigger for the alternative vowel height (see simulated KWS performance for Subject 1 in Supplementary Material). These observations demonstrate why whole-word keyword spotting approaches are better suited for neural data. Some phonemic representations may not be sampled by a particular electrode coverage even with high-density spatial sampling—particularly if the neural populations associated with those articulators are located in a sulcus, which surface ECoG has difficulty sampling. They further demonstrate the inherent difficulty of performing phonemic-based automatic speech recognition ala (Herff et al., 2015) using even the high density neural recordings from our best-performing subjects.

Critically, the results of this study suggest that the precise temporal sequencing of neural activity correlated with the subset of neural articulator representations that *are* sampled can be sufficient to discriminate a keyword from non-keyword speech. Furthermore, neural keyword spotting has several significant advantages to an acoustic keyword spotting system. Neural keyword spotting is capable of activating selectively to the intended speaker even in the presence of multiple speakers, and it performs keyword discrimination using features that can discriminate acoustically similar words like “Alexa” and “Balexa” which most commercial keyword spotting systems would struggle with using acoustic features alone—especially in the presence of noise. Although utterance detection using the peak-picking algorithm described in this study would likely need modification to properly trigger for keywords that occur mid-vocalization, our results suggest that neural VAD as described by this study would perform well for interaction with a virtual agent, wherein a period of silence is followed first by the keyword, then the command for the agent. We further propose that our results demonstrate encouraging performance that motivates a follow-up study using practical keywords in a less constrained scenario involving continuous speech.

## 5. Conclusions

This study suggests that a high-sensitivity/specificity one-bit neural keyword spotting BCI can be created using ECoG recordings from vSMC and STG. Neural signals capturing speech motor representations from vSMC appear to be useful for low-latency (~1 s) and high-specificity VAD, while a combination of neural signals from vSMC and auditory representations from STG may be useful for discriminating keyword utterances from non-keyword speech. Spatiotemporal relationships of high gamma activity across electrodes, captured and efficiently quantified using a method of neural template correlation, appear to be instrumental for keyword discrimination. In this study, keyword-spotting performance depended on several factors including electrode density and the number of electrodes within vSMC and STG. Our results suggest that high-density ECoG grids may be necessary and sufficient for capturing the spatial layout of cortical speech representations needed for a keyword-spotting neural interface. Neural features that provide information about consonant articulation appear to be best represented in vSMC, with place of articulation primarily encoded by the spatial location of high-gamma activity and consonant voicing encoded by the temporal dynamics of this activity. Vowel height during overt speech appeared to be poorly encoded by vSMC, but better represented in traditionally auditory areas along STG during self-monitoring. Although we did not test whether neural activity in STG during covert speech was sufficient for decoding vowel height, other studies have indicated that this may be possible (Pei et al., 2011b; Leuthardt et al., 2012). Together with these and other studies, our findings support the feasibility of keyword spotting with an ECoG BCI provided that relevant cortical areas are recorded with sufficient spatial sampling and that keywords are composed of neurally discriminable articulatory gestures.

## Data Availability

The datasets generated and analyzed for this study, analysis code, and latex source files for this paper can be found in the gigantum project located at: https://gigantum.com/griffinmilsap/ecog-keyword-spotting. Stimulus parameters for BCI2000 and BCI2000 Web are available at https://github.com/cronelab/SyllableTasks.

## Author Contributions

MC, YW, and NC designed the experiment and utterance contrasts. GM, MC, CC, and YW collected the intracranial recordings. GM conceived and conducted analyses. MC, QR, YW, and NC provided feedback and direction. GM authored the manuscript. GM, MC, CC, QR, YW, and NC reviewed the manuscript and associated submission documents.

### Conflict of Interest Statement

The authors declare that the research was conducted in the absence of any commercial or financial relationships that could be construed as a potential conflict of interest.
