# Supplementary material for: Keyword Spotting Using Human Electrocorticographic Recordings
Source: Front Neurosci. 2019 Feb 19;13:60. doi: 10.3389/fnins.2019.00060 (PMC6389788; doi:10.3389/fnins.2019.00060)
Supplement: Supplementary file 1 [file Data_Sheet_1.pdf]

Supplementary Material: Keyword Spotting using Electrocorticographic Recordings

1 SUPPLEMENTARY TABLES AND FIGURES

1.1 Figures

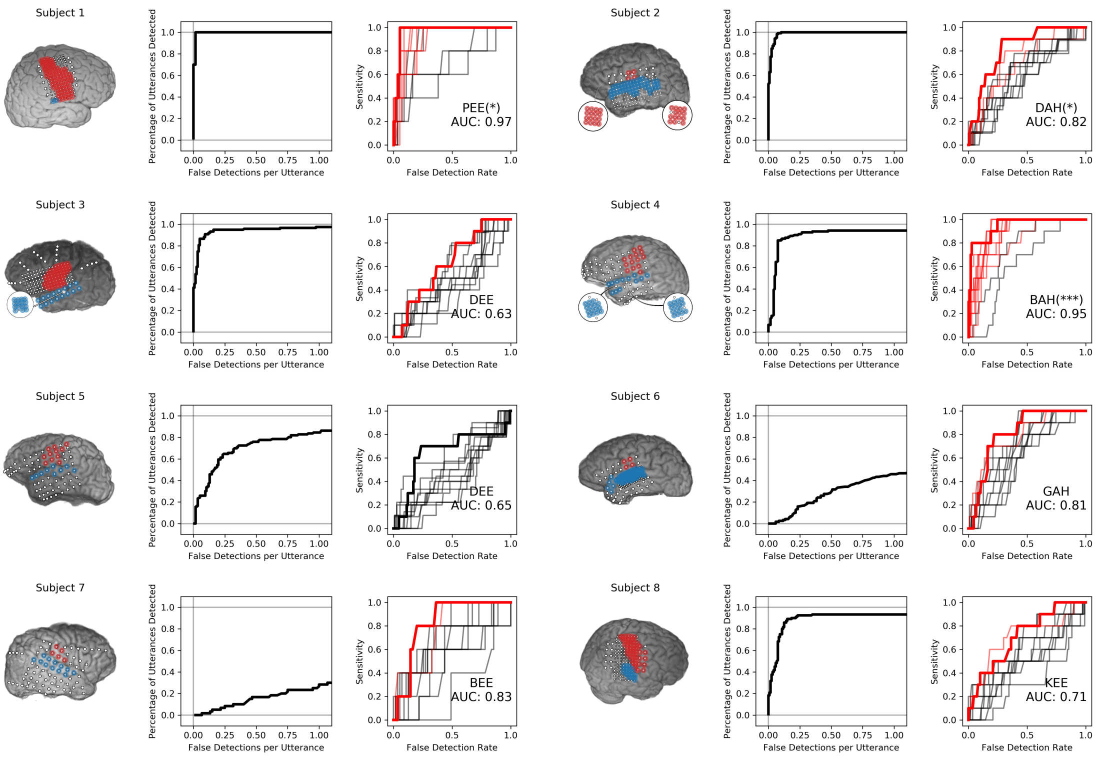

Figure S1. Isolated VAD and keyword ROCs for all subjects. The left-most panel shows the electrodes highlighted in red and blue that were used to discriminate syllables. Only electrodes highlighted in red were used to perform VAD. The center panel shows the VAD performance in sensitivity (percentage of utterance timings correctly identified) against the number false detections per utterance for various VAD thresholds. The right-most panel shows ROC curves for all twelve keyword detectors. ROC curves with AUC values were significant at the 95% confidence interval are highlighted in red. The keyword detector that produced the highest AUC is highlighted in bold-red and indicated via annotation under the curves, followed by asterisks indicating significance at the $p < 0.05$ (*), $p < 0.01$ (**) and the $p < 0.001$ (***) level with respect to the distribution of maximum AUC models.

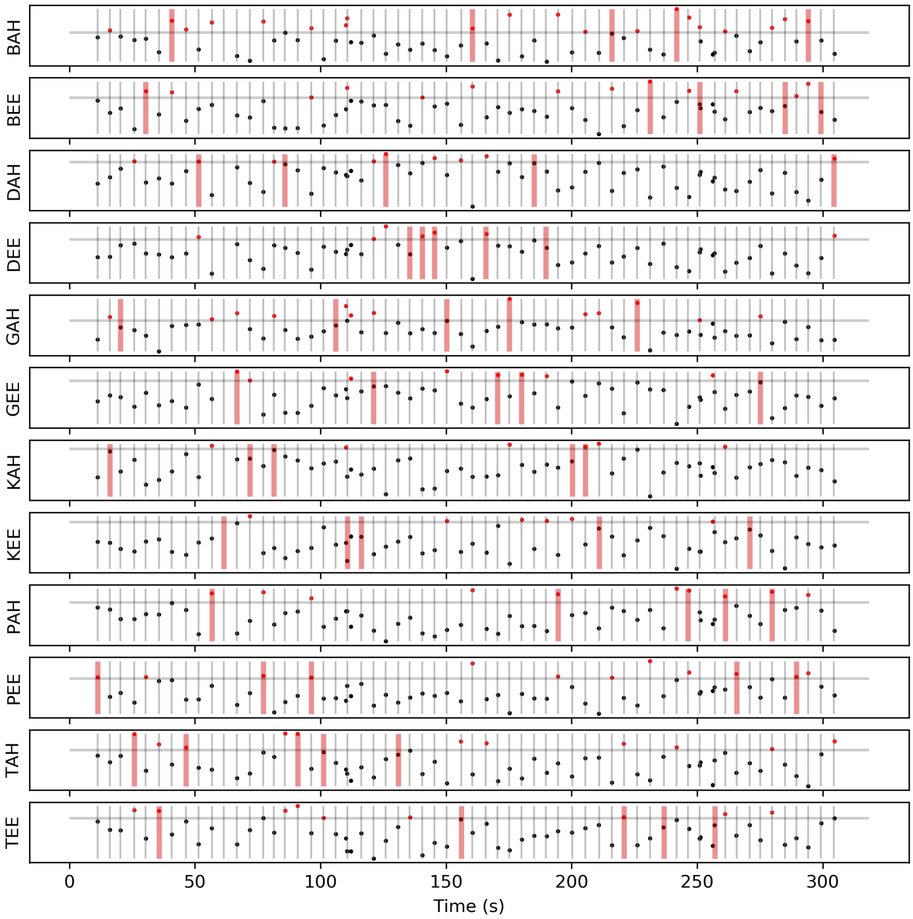

Figure S2. Simulated real-time performance for Subject 1 across the repetition task. Performance for models trained to identify one keyword vs non-keyword speech is visualized for every one of the twelve keyword models. Ground truth utterances are denoted by vertical lines, and utterances that the model should identify as "keyword" utterances are highlighted in bold red. Times during which the VAD model identified utterances are indicated with black dots, the Y-value of these dots indicates a unit-less classifier output from the features at the time of the detection; higher placement indicates a more "keyword-like" utterance. Each keyword spotter has had a threshold of discrimination set at 0.75 the equal error rate, so as to promote higher sensitivity at the expense of more false-detections. Super-threshold classifier output is highlighted with a red dot rather than a black one, indicating the detection of a keyword.

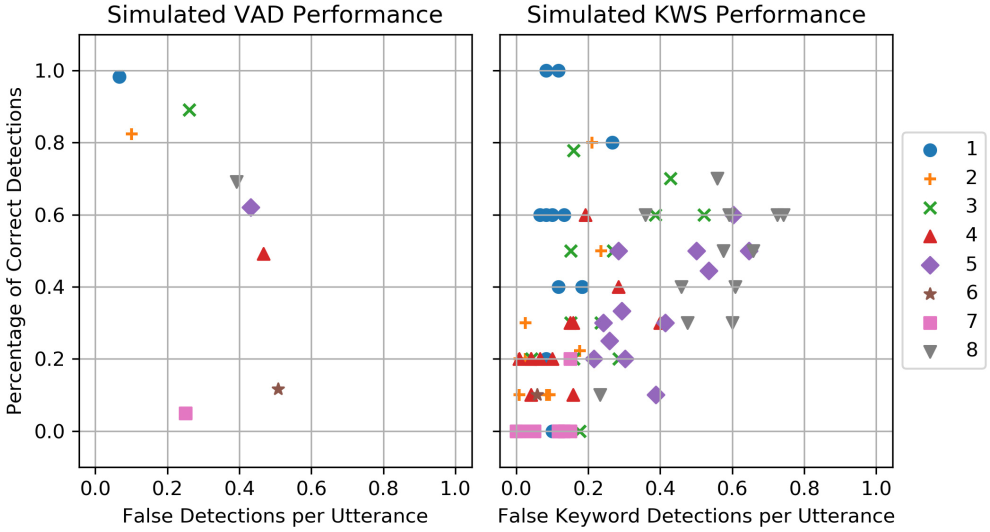

Figure S3. Simulated VAD and KWS performance on the testing dataset. On the left, the percentage of utterances correctly detected by neural VAD is plotted against the number of false utterance detections for all subjects in the study. On the right, performance for all twelve keyword spotters from each patient are plotted.
